# Blepharoplasty Online: Critical Analysis of Content and Patient Comprehensibility

**DOI:** 10.1007/s00266-024-04083-1

**Published:** 2024-05-24

**Authors:** Joseph Kaleeny, Emma Levine, Lauren Okamoto, Shayan A. McGee, Jeffrey E. Janis

**Affiliations:** 1 Department of Plastic and Reconstructive Surgery, The Ohio State University Wexner Medical Center, 915 Olentangy River Rd, Columbus, OH 43210 USA; 2 Section of Surgical Sciences, Department of Surgery, Vanderbilt University Medical Center, Nashville, TN USA; 3 Department of Surgery, Robert Larner, MD, College of Medicine at the University of Vermont, Burlington, VT USA; 4 Department of Surgery, Dartmouth Medical School, Hanover, NH USA

**Keywords:** Blepharoplasty, Readability, Online patient education

## Abstract

**Introduction:**

Patients frequently turn to online information for decision-making factors about aesthetic procedures. The quality of online medical content is an essential supplement to clinical education. These resources assist patients in understanding the risks, benefits, and appropriateness of their desired procedure. This study examines the breadth and readability of online blepharoplasty information, elucidating its educational utility.

**Methods:**

A depersonalized Google search was conducted using the Startpage Search Engine, investigating key phrases, “blepharoplasty decision making factors”, “eye lift decision making factors”, and “eyelid lift decision making factors”. The first three pages of results for each search term, totaling 90 links were screened. Data were extracted for various decision-making factors, subspecialty, gender, and readability.

**Results:**

Twenty-six websites met inclusion for analysis. Thirteen websites were plastic surgery based, five otolaryngology (ENT), five ophthalmology/oculoplastic, one oral-maxillofacial (OMFS), and two mixed-based practices. Most blepharoplasty webpages identified were that of private practice and male surgeons. Half were subspecialties other than plastic surgery. Thirteen common decision-making factors were identified. The most common factors addressed across all texts were recovery followed by cosmetic and functional goals. The least discussed were genetic factors. Average Readability exceeded the 12th grade. There were no significant differences in readability means among subspecialties.

**Conclusion:**

This study examines the online blepharoplasty sphere among US-based practices providing clinical education to patients. No appreciable differences among gender, subspecialty, and readability on decision-making factors were found, highlighting a consistency among surgeons. Most websites fell short of readability standards, however, emphasizing a need for clearer information to patients.

**No Level Assigned:**

This journal requires that authors assign a level of evidence to each submission to which Evidence-Based Medicine rankings are applicable. This excludes Review Articles, Book Reviews, and manuscripts that concern Basic Science, Animal Studies, Cadaver Studies, and Experimental Studies. For a full description of these Evidence-Based Medicine ratings, please refer to the Table of Contents or the online Instructions to Authors www.springer.com/00266.

## Introduction

Blepharoplasty is the fourth most common cosmetic surgery performed in the USA, with 115,261 procedures performed in 2022, a 13% increase from 2019 [1]. Blepharoplasty is widely performed across various qualified professionals including plastic surgeons, otolaryngologists (ENT), ophthalmologists, oculoplastic surgeons, and oral-maxillofacial surgeons. University and private practice-based webpages serve as platforms on which surgeons can disseminate detailed information about procedures, showcase before-and-after photographs, and convey outcomes and potential complications to patients.

Online medical information has become a vital resource for patients seeking knowledge about desired surgical procedures. One study assessing aesthetic surgery patients found that 95% of patients rely on the Internet to research procedures prior to in-office consultations [2]. Another study utilized patient-administered questionnaires found that over half of these patients endorse using plastic surgery websites to find information [3, 4]. While web-based platforms offer significant potential for patient education, a consistent limitation of plastic surgery websites has been readability [5]. A recent study evaluated the quality of printed online educational materials in cosmetic facial plastic surgery including blepharoplasty to assess reliability using the DISCERNs score, a tool used to evaluate the quality and reliability of written health information. Findings indicated the quality of printed online education materials pertaining to elective facial cosmetic surgery were “fair,” corresponding to a quantitative range between 42 and 54 out of a total score of 80 [6]. Overall, many webpages are above the recommended sixth-seventh grade reading level recommended by the American Medical Association and the National Institutes of Health. [7–10]

In aesthetic surgery, making well-informed decisions is paramount for patients. Patients meticulously weigh factors such as their overall health, anticipated recovery times, and the experience and expertise of the surgeon [11–13]. Although previous studies have shown that the gender of the surgeon may not influence decision-making, this is often a topic of conversation [14, 15]. In parallel, surgeons must address individual patient functional and aesthetic goals, navigating treatment opportunities and managing challenging expectations [16]. By thoroughly researching and understanding these factors, patients can confidently embark on their blepharoplasty journey.

Ultimately this study aims to identify decision-making factors in the online blepharoplasty space while addressing readability for patients seeking medical recommendations. Given the diversity of specialties performing this procedure, the study also aims to assess any differences in online content between specialties. Finally, this study assesses differences by surgeon gender to determine if online resources echo trends in decision-making identified in prior studies [14, 15].

## Methods

### Webpage Identification

Backlinko’s most recent Search Engine Optimization Organic-Click-Through-Rates report (published May 28th, 2023) was used to determine the appropriate number of results to analyze and identified the first 30 results or three pages from each search term. The first result on Google received 27.6% of all consumer clicks, while only 0.63% of Google searchers clicked on links from the second page [17]. The first three pages of Google results, comprising 30 listings without sponsorship, cover nearly 100% of consumer visibility with 10 results per page. Ninety total links were recorded from the three individual search terms. Additionally, to depersonalize search results, Startpage.com was utilized to send anonymous, depersonalized searches to Google’s search engine that are not influenced by user settings, location, IP address, search history, or cookies [18].

Terms “Blepharoplasty decision making factors”, “eye lift decision making factors”, and “eyelid lift decision making factors” were searched using Startpage.com on October 5th, 2023 to capture user intent. In search engine optimization, user intent is algorithmically considered, resulting in comparable outcomes from similar or slightly varied search terms, despite potential differences in terminology. This methodology and choice of search terms were adapted from Fanning JE, et al.’s 2023 paper, ‘Content and Readability of Online Recommendations for Breast Implant Size Selection’ due to its relevance to our objectives and established efficacy [19]. The first 30 results, or three pages from each search term, were screened. Inclusion criteria were United States (US) medical-based webpages related to blepharoplasties. Exclusion criteria were (1) advertisements or sponsorships, (2) non-US-based webpages, and (3) nonpractice webpages such as those belonging to information sites, or companies. Sixty-three nonrelevant and or duplicate pages were removed. Twenty-six unique webpages were selected and examined for data extraction.

### Webpage Text Qualitative Analysis

Webpage texts were isolated and analyzed independently by a single author (J.K.) for commonly discussed decision-making factors of blepharoplasties. Four broad-based categories were identified including patient characteristics, pre-procedure considerations, surgeon input, and surgical elements. These categories were further subdivided into 13 unique descriptors. Webpages were also assessed for academic or private practices, surgeon’s gender, surgical subspecialty, and US state location.

### Webpage Text Readability Analysis

Webpage texts were batch-imported into Readability Professional Studio software [20]. Automated Readability Index, Bormuth Grade Placement, Coleman-Liau (grade levels), Degrees of Reading Power (grade equivalent), Flesch-Kincaid, Fry, Gunning Fog, Harris-Jacobson Wide Range Formula, LIX (grade levels), New Dale-Chall, RIX (grade levels), Raygor Estimate, and SMOG measures were run by batch calculation on October 10, 2023. In addition, webpage texts were imported into the Hemingway Editor webtool [21]. Readability results and text metrics were extracted for analysis.

## Results

### Practice Breakdown

**Fig. 1:**
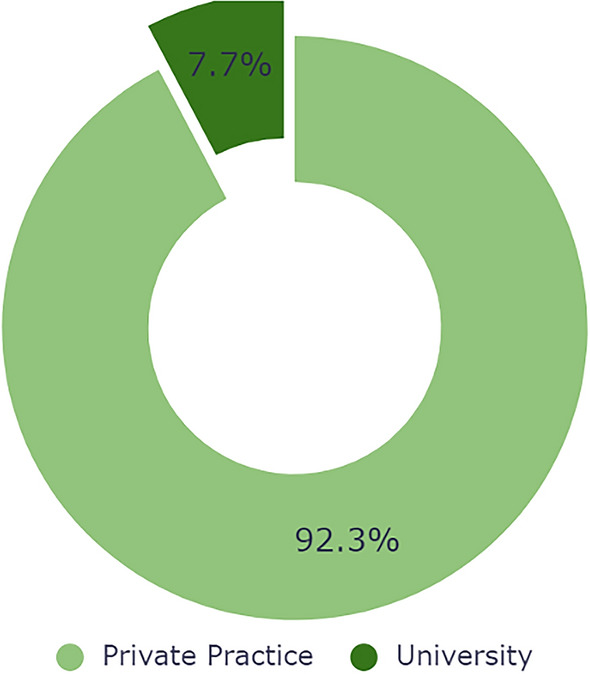
Private vs university United States-based practices

**Fig. 2:**
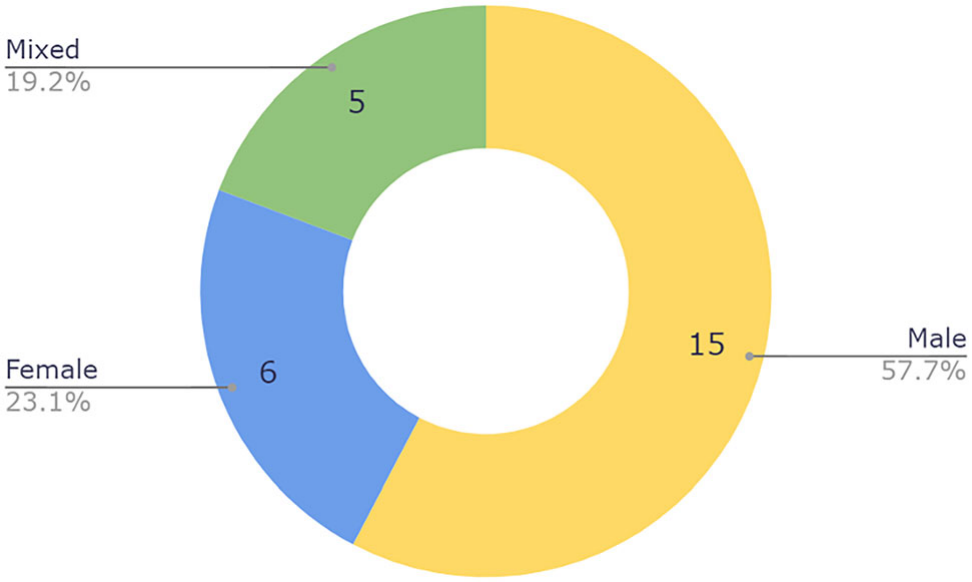
Gender breakdown among practices

**Fig. 3.**
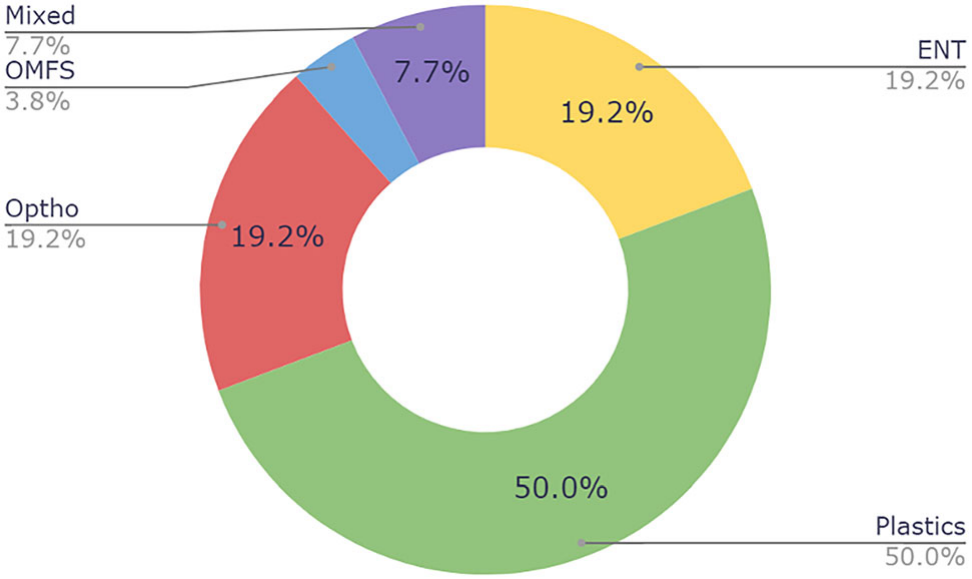
Specialty breakdown among practices

**Fig. 4:**
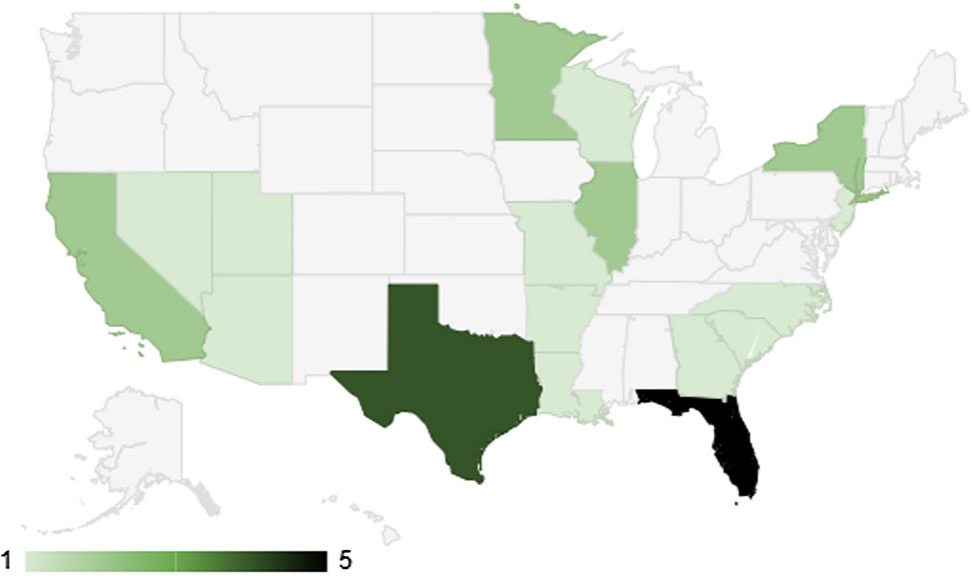
Visual map of practice distribution

Twenty-four practices were private, the remaining two were academic/university-based, both visually represented in Fig. 1. Most practices had only male surgeons (57.7%). Only 23.1 % of practices were comprised of female-only providers, while 19.2% were staffed by both men and women (Fig. 2). ENT represented a 4:1:0 ratio of men to women to mixed practices, plastics 8:2:1, ophthalmology/oculoplastic 2:1:2, oral-maxillofacial 1:0:0, and mixed practices 0:0:2. Most webpages were plastic surgery based at 50.0%, 19.2% by otolaryngologists, 19.2% by ophthalmologists or oculoplastic surgeons, 3.8% by oral-maxillofacial, and 7.7% of practices with multiple specialties (Fig. 3). Practices were distributed across 17 US states, Florida and Texas being the most frequent (Fig. 4).

### Decision-Making Factors

The most common decision-making factor identified in online resources was recovery (100.0%) and the least common decision-making factors discussed were genetic factors/ethnicity (23.8%), followed by longevity and past medical history (50.0% and 61.54% respectively). Table 1 provides an overview of the 13 identified decision-making factors and their prevalence among webpages.

**Table 1:** Decision-making factors referenced and discussed among webpages

Broad categories	Decision-making factors identified	Percentage of webpages	Example considerations
Patient based	Past medical history	61.54%	Overall good health
	Patient characteristics	73.08%	Age
	Genetic factors / ethnicity	23.08%	Hereditary factors
	Facial structure	80.77%	Eyelid position
Pre – procedure considerations	Realistic expectations	61.54%	Expectation for results that can be obtained
	Cosmetic goals / concerns	88.46%	Improve excess skin on upper/lower eyelid
	Functional concerns / goals	88.46%	Vision difficulties
Surgeon based	Surgeon input and authority	65.38%	Surgeon maintains patient safety and satisfaction
Surgery based	Recovery	100%	Downtime after surgery
	Risks & complications	80.77%	Risks such as dry/irritated eyes
	Surgery explanation	80.77%	Upper blepharoplasty - what that addresses vs lower blepharoplasty; description of the surgery
	Cost / insurance coverage	84.62%	Insurance vs out-of-pocket considerations
	Longevity of results	50.00%	How long results may last

### Comparative Analysis

A two-way ANOVA was conducted to assess the impact of gender and subspecialty on decision-making factors. The analysis revealed a non-significant main effect of gender (*F*(2, 16) = 0.073, *p* = 0.93) and subspecialty (*F*(4, 16) = 1.499, *p* = 0.249). Additionally, there was no significant interaction between gender and subspecialty (*F*(3, 16) = 0.602, p = 0.623).

The overall model was also not statistically significant (*F*(9, 16) = 1.415, *p* = 0.261). Post-hoc analyses did not reveal any significant differences between specific groups. The *R*-squared value for the model was 0.443, indicating that approximately 44.3% of the variance in the decision-making factor can be explained by the combined influence of gender and subspecialty. The adjusted *R*-squared value, which accounts for the number of predictors in the model, was 0.130. The results suggest that neither gender nor subspecialty significantly influenced decision-making factors in the sample. Interaction between gender and subspecialty also did not significantly affect decision-making factors.

### Readability Analysis

**Fig. 5:**
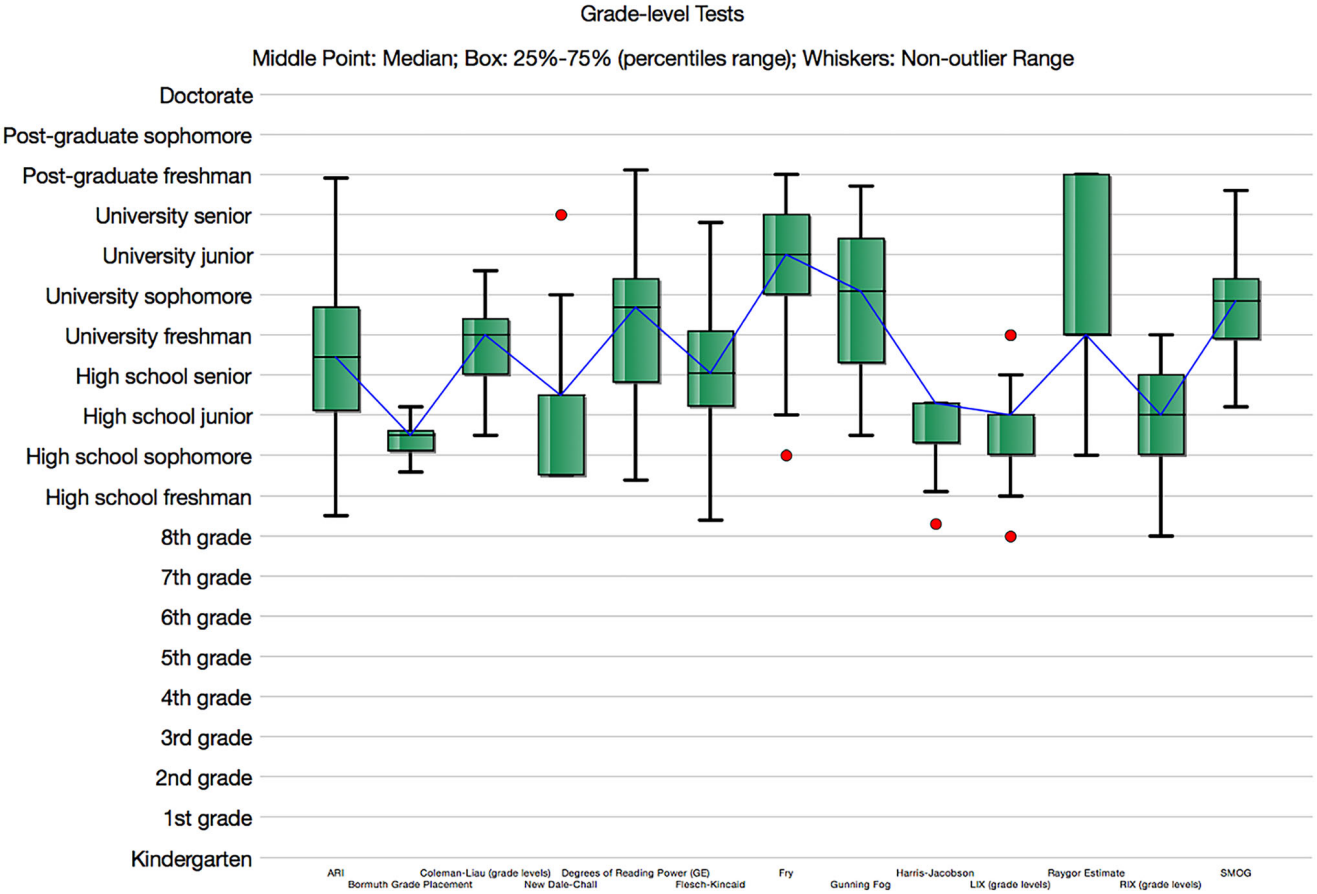
Grade-level tests across 13 unique readability measures

**Fig. 6:**
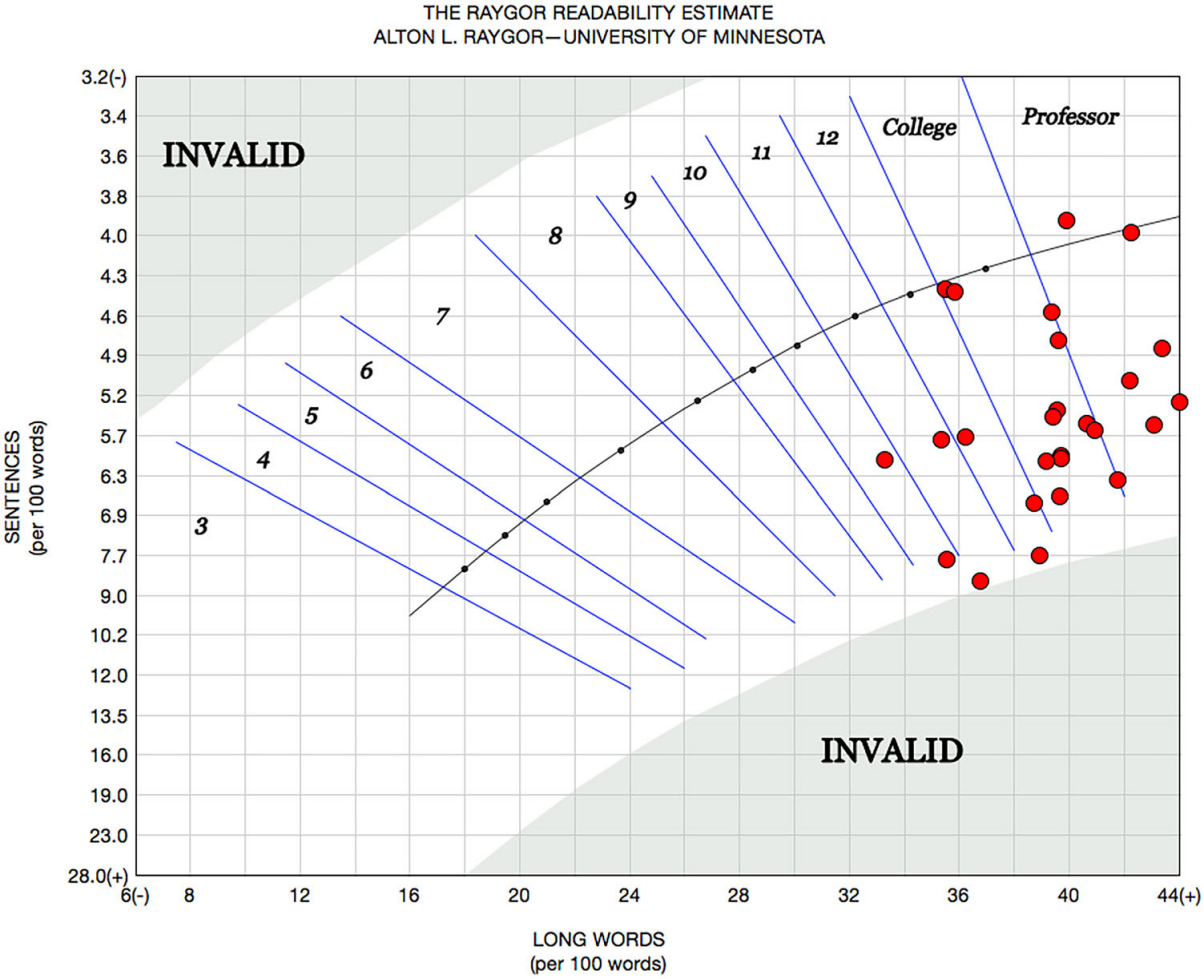
Raygor readability estimates

**Fig. 7:**
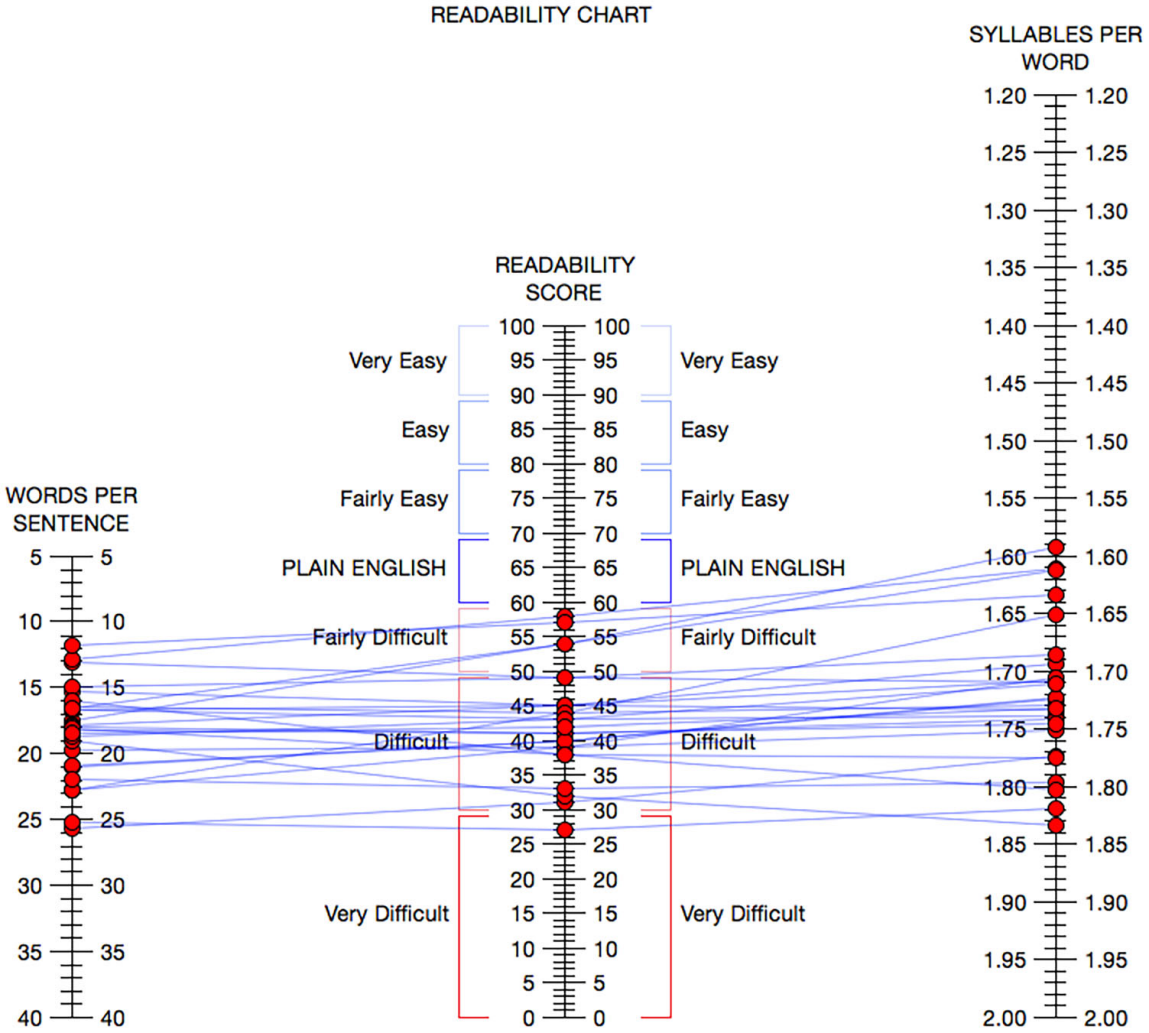
Flesch reading ease readability

**Fig. 8:**
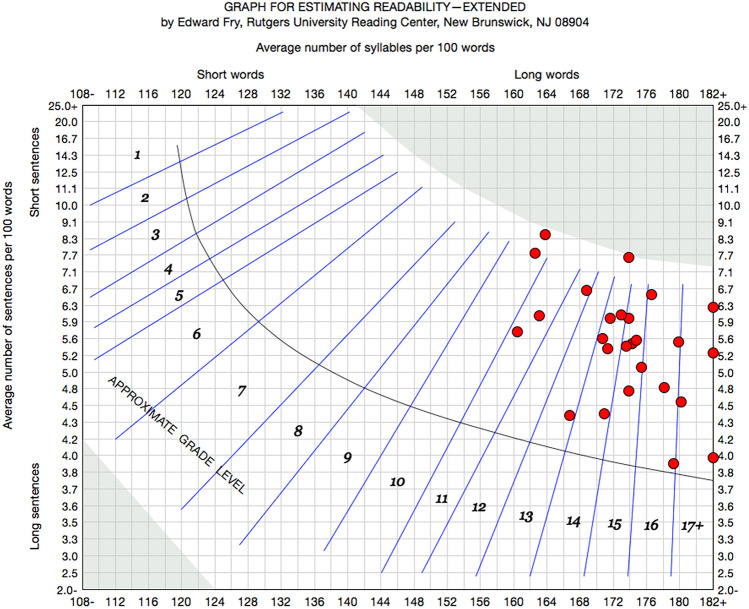
Fry readability scores

The range of readability scores spanned from the 8th to 16th (university senior grade level) across all readability metrics. Several webpage readability scores extended beyond that of college grades. Table 2 lists mean Grade-level Test scores across all 13 readability measures utilized in this study and Fig. 5 illustrates these results. Across all readability measures the mean grade level was 12.4. Figure 6 provides an illustration of the Raygor Readability Estimates across all webpages demonstrating mean scores at the university sophomore level. Line correlation between words per sentence, readability score, and syllables per word is demonstrated in Fig. 7, the Flesch Reading Ease Readability Chart, with most webpage texts crossing the “difficult” to “fairly difficult” range. Moreover, Fig. 8 provides the distribution of individual webpage Fry Readability scores, mean score as grade 14, or university sophomore. Of note, the average educational grade level requirement comprehension exceeded the 8th grade level, Table 3 provides the full report of the average webpage readability scores from the Hemingway Editor.

**Table 2:** Readability means scores of webpages

Readability professional studio software		
Readability measure	Average readability score	Scale
Automated Readability Index	12.3	Grade level
Bormuth Grade Placement	10.4	Grade level
Cloeman-Liau (grade levels)	12.7	Grade level
Degrees of Reading Power (grade equivalent)	13.3	Grade level
Flesch-Kincaid	12	Grade level
Fry	15	Grade level
Gunning Fog	14	Grade level
Harris-Jacobson Wide Range Formula	10.8	Grade level
LIX (grade levels)	11	Grade level
New Dale Chall	11.6	Grade level
RIX (grade levels)	10.6	Grade level
Raygor Estimate	14	Grade level
SMOG	13.8	Grade level

**Table 3:** Hemingway Editor averages of webpages

Hemingway editor writing analysis webtool	
Textual analysis elements	Average score
Education required to read (grade level)	8.9
Reading time	6 minutes and 14 seconds
Number of words	1563.3 words
Number of sentences	141.3 sentences
Number of uses of passive voice	17.5 uses of passive voice
Number of phrases with simpler alternative	10.5 phrases with simpler alternatives
Number of sentences that are hard to read	18.5 sentences that are hard to read
Number of sentences that are very hard to read	24.0 sentences that are very hard to read

A one-way ANOVA test revealed no significant differences in Readability Measures across the five subspecialties (*F*(4, N) = 1.180, *p* = 0.348). This suggests that, in our study's context, subspecialties do not have a statistically significant impact on the readability of the provided materials. Table 4 outlines mean readability scores per subspecialty.

**Table 4:** Mean readability scores per specialty

Average readability means per specialty	
Specialty	Readability means (std. deviation)
ENT *n* = 5	11.6 (1.8)
Plastics *n* = 13	12.7 (1.5)
Ophthalmology/Oculoplastic *n* = 5	12.4 (0.9)
Oral Maxillofacial Surgery *n* = 1	13.1
Mixed *n* = 2	10.7 (1.8)

## Discussion

In this study, we investigated the online blepharoplasty space for common decision-making factors and their relationship with gender and subspecialty of the surgeon within webpages corresponding to practices in the USA. Our findings provide valuable insights into the diversity of practices and the factors influencing decision-making processes in this field.

The distribution of practices in our study revealed an interesting landscape within the plastic and reconstructive surgery domain for blepharoplasty. Most practices were private, with only a small proportion associated with academic or university settings. Practices led solely by male surgeons were predominant, which was expected given the male to female ratio reported by the American Society of Plastic Surgeons of 5:1 [22]. Geographically, our results suggested that practices performing blepharoplasty were spread across 17 states, with Florida and Texas emerging as the most common locations. These results differ slightly from a 2013 publication that reported on all aesthetic procedures in the USA with the Mountain/Pacific region reporting 30% of all procedures followed by New England/Middle Atlantic and the South Atlantic at 19%, East/West South Central and 5th East/West North Central at 16% [23].

Decision-making factors identified and discussed on practice websites offer a glimpse into the considerations that patients might encounter when seeking plastic and reconstructive surgery services. Recovery emerged as the overwhelmingly prevalent factor, indicating its universal importance to patients. On the other end of the spectrum, genetic factors and ethnicity were discussed least frequently, suggesting a potential gap in addressing personalized patient concerns. Longevity and past medical history, while more commonly discussed than genetic factors, still represented only half and slightly over half of the practices, respectively. This discrepancy in emphasis on different factors underscores the need for further exploration into how surgeons approach discussions about these variables with their patients.

The comparative analysis of decision-making factors with respect to surgeon gender and subspecialty provided intriguing insights. Our results demonstrated that neither surgeon gender nor subspecialty significantly influenced the decision-making factors discussed on the blepharoplasty information websites. The absence of significant differences between specific groups indicates a consistent approach online to patient communication regardless of the surgeon's gender or subspecialty. In corroboration, a 2020 paper identified that through patient satisfaction surveys, the satisfaction scores of surgeons are more closely correlated with patient variables than surgeon factors, including gender [24]. These findings may challenge preconceived notions about potential gender-based or subspecialty-related biases in patient communication within the plastic and reconstructive surgery field. Ultimately, however, the study focused on a limited number of websites, which may have posed challenges in achieving statistical significance due to the small sample size. With only a small subset of webpages analyzed, particularly in relation to gender and subspecialty, the findings may not fully reflect the broader landscape of online information regarding blepharoplasty.

For all webpages, the mean readability score was above the 12th grade level, exceeding the sixth-seventh grade reading level recommended by the American Medical Association and National Institutes of Health for publicly available health-related information [7, 25]. These findings present a fundamental issue in patient comprehension considering the average American adult reads at an eighth-grade level [26, 27]. Approximately, 47% of adults in the United States “experience considerable difficulty in performing tasks that required them to integrate or synthesize information from complex or lengthy texts” [28]. Furthermore, it was observed that the readability of the webpages remained consistent across various specialties and the gender of the providers, indicating only marginal differences. These results underscore the importance of careful assessment of the readability level and content of webpages for healthcare professionals, particularly those in plastic surgery, to optimize comprehension by prospective patients and augment pre-operative education.

While this study focused on webpage content, the evolving role of social media platforms such as Instagram, X (formerly known as Twitter), and TikTok as significant sources of information, particularly for younger patients, continues to grow. Incorporating data from these photo and video-based platforms could yield valuable insights into patient perspectives on aesthetic procedures. Examining marketing tactics using metrics like online traction and referrals from social media platforms could offer a more comprehensive understanding of their influence on online patient education and dissemination. This contributes to a broader perspective on patient information-seeking behavior and clinical practice enhancement.

Based on the results of this study, the study team has identified several suggestions to optimize web-based materials for patient comprehension:

1. Focus on Recovery: Given universal importance to patients, dedicating a specific section to the recovery process post-blepharoplasty could be highly beneficial. Recommended topics include anticipated recovery times, what patients can expect during this period, and how to facilitate a smooth recovery.
2. Address Genetic Factors and Ethnicity: The study highlighted a gap in discussions related to genetic factors and ethnicity. Including information on how these factors might influence or relate to blepharoplasty outcomes could add a valuable dimension to patient education.
3. Address the brow and midface positioning: Although not highlighted explicitly in this review, it’s relation to the eyelid appearance is crucial and warrants attention for comprehensive patient education.
4. Highlight Longevity and Past Medical History: While these factors were discussed in many practices, further elaborating on the impact in blepharoplasty outcomes or candidacy could enhance patient understanding.
5. Emphasize Surgeon Expertise and Experience: As patients often consider the experience and expertise of the surgeon, featuring detailed profiles of the surgeons involved in performing blepharoplasty procedures could instill confidence and trust in potential patients.
6. Readable Content: A crucial aspect highlighted by the study is the readability of the information provided. Suggestions would include simplifying complex medical jargon, using clearer language, and structuring content that is easier for patients to comprehend, ideally aiming for a reading level closer to the sixth-seventh grade as recommended by health authorities.
7. Visual Aids and Multimedia: Including before-and-after photos, videos explaining the procedure, and infographics illustrating key points can significantly enhance patient education and comprehension.
8. Patient Testimonials or Stories: Sharing experiences of past patients who underwent blepharoplasty could offer insights and alleviate concerns for prospective patients.

## Conclusion

This study provides valuable insights on the varied practice features and decision-making factors pertinent to blepharoplasty within the realm of online plastic and reconstructive surgery practices. By identifying gaps in written patient communication, the research highlights areas for improvement, emphasizing the need for clearer, more comprehensive information on plastic surgery websites. Our findings reveal that many blepharoplasty webpages still fall short of national readability guidelines, indicating the urgency for enhanced content and readability. It is important to note the limitations inherent in this research, including its focus on websites, which do not fully capture the intricacies of patient-surgeon interactions. Future studies should explore other popular aesthetic procedures, employ qualitative methodologies to a broader range of variables, and potentially expand internationally. These would enable a comprehensive understanding of global patterns in online health information accessibility and credibility.
